# Flower-like supramolecular self-assembly of phosphonic acid appended naphthalene diimide and melamine

**DOI:** 10.1038/srep14609

**Published:** 2015-09-29

**Authors:** Rajesh S Bhosale, Mohammad Al Kobaisi, Sidhanath V. Bhosale, Suresh Bhargava, Sheshanath V. Bhosale

**Affiliations:** 1 RMIT-IICT Research Centre, CSIR-Indian Institute of Chemical Technology, Hyderabad- 500 007, Telangana, India; 2 School of Applied Sciences, RMIT University, GPO Box 2476, Melbourne, Vic. 3001, Australia; 3 Polymers and Functional Materials Division, CSIR-Indian Institute of Chemical Technology, Hyderabad-500 007, Telangana, India; 4 Centre for Advanced Materials and Industrial Chemistry (CAMIC), RMIT University, GPO Box 2476, Melbourne, Vic. 3001, Australia

## Abstract

Diverse supramolecular assemblies ranging from nanometres to micrometers of small aromatic π-conjugated functional molecules have attracted enormous research interest in light of their applications in optoelectronics, chemosensors, nanotechnology, biotechnology and biomedicines. Here we study the mechanism of the formation of a flower-shaped supramolecular structure of phosphonic acid appended naphthalene diimide with melamine. The flower-shaped assembly formation was visualised by scanning electron microscope (SEM) and transmission electron microscopy (TEM) imaging, furthermore, XRD and DLS used to determined mode of aggregation. Characteristically, phosphonic acid-substituted at imide position of NDIs possess two important properties resulting in the formation of controlled flower-like nanostructures: (i) the aromatic core of the NDI which is designed to optimize the dispersive interactions (π**-**π stacking and van der Waals interactions) between the cores within a construct and (ii) phosphonic acid of NDI interact with malamine through molecular recognition i.e. strong hydrogen-bonding (H-bonding). We believe such arrangements prevent crystallization and favour the directional growth of flower-like nanostructure in 3D fashion. These works demonstrate that complex self-assembly can indeed be attained through hierarchical non-covalent interactions of two components. Furthermore, flower-like structures built from molecular recognition by these molecules indicate their potential in other fields if combined with other chemical entities.

Diverse supramolecular assemblies ranging from nanometres to micrometers of small aromatic π-conjugated functional molecules have attracted enormous research interest in light of their applications in optoelectronics, chemosensors, nanotechnology, biotechnology and biomedicines^1^,^2^,^3^,^4^,^5^,^6^. Weak molecular non-covalent forces such as hydrogen-bonding, π**-**π interaction, van der Waals interaction and electrostatic interaction play important roles in the construction of controlled one-dimensional (1D), two-dimensional (2D) and three-dimensional (3D) supramolecular structures such as nanowires, nanoribbons, nanotubes, nano/micro-belts, nanosheets, and nano/micro-flowers from small aromatic π-conjugated functional molecules^7^,^8^,^9^,^10^,^11^,^12^. Although the massive morphological changes of supramolecular assemblies have been widely investigated few works addressed the two-component self-assembly^13^,^14^,^15^,^16^,^17^,^18^, which offer controlled assembly method for producing versatile soft matters from simple organic components. Amongst two molecule self-assembly, melamine (MM) is one of the most fascinating scaffolds in supramolecular chemistry possess hydrogen bonding donor as well as acceptor sites. MM and its derivatives mediated supramolecular self-assemblies of small aromatic π-conjugated molecules have been studied for the construction of nano/micro-meter soft functional materials^13^,^18^.

During the past few years flower-shaped inorganic^19^,^20^,^21^,^22^,^23^/organic^24^,^25^,^26^,^27^,^28^,^29^,^30^,^31^ assemblies gained attention of researchers because of their potential applicability in the field of catalysis, superhydrophobic (non-wetting) materials, explosives detection materials, magnetic materials, biomedical materials and optoelectronic materials^19^,^20^,^21^,^22^,^23^,^24^,^25^,^26^,^27^,^28^,^29^,^30^,^31^. Recently, the first organic flower-shaped morphology *via* self-organization of functionalized C_60_ derivative was reported by Nakanishi and co-workers^32^. Furthermore, benzothiophene derivatives, nucleoside derivatives, diphenylalanine dipeptide and DNA are also utilized by various research groups for the construction of organic flower-like nanostructures^24^,^25^,^26^,^27^,^28^,^29^,^30^,^31^. In the view of literature survey it reveals that the organic flower-shaped morphology filed is in its infancy and has not been explored more in terms of various small aromatic π-conjugated functional molecules.

To further explore this idea of small aromatic π-conjugated molecules naphthalene diimide (NDI) was chosen as one of the counter components for two molecules self-assembly study. As NDI is a compact, planar, aromatic and most fascinating π-conjugated compound it has been widely studied. Subsequently it has found use in organic electronics because it has excellent n-type conductivity and stability. Its ability to self-assemble with various functionalities such as amino acids, peptides, pyridyl, viologen derivatives, hydrophilic as well as hydrophobic aliphatic chains for the construction of supramolecular assemblies such as nanotubes, nanobelts, nanoparticles, organogels, hydrogels, synthetic ion channels and also used in various larger multicomponent assemblies^32^,^33^,^34^,^35^,^36^,^37^,^38^,^39^,^40^,^41^,^42^,^43^.Recently we explored two examples that have been used for self-assembly which comprised of phosphonic acid either on one or both sides of the NDI’s through dimide nitrogen^44^,^45^. The pH-dependent self-assembly one-sided phosphonic acid NDI produced well defined interwoven fibres, whilst a ladder-type network was observed at neutral pH and at basic pH it assembled into more complex fractal nanostructures^44^. Alternatively, NDI functionalised with phosphonic acid on the both side of imide position self-assembled with L- and D-arginine through chirality induced molecular recognitions and led to the formation of micrometre long nanobelts and spherical aggregates at pH 9 in water, respectively^45^. We hypothesise that the expansion of the planar aromatic core of NDI can be employed to construct novel nanostructures by strengthening π**-**π-stacking and spontaneous self-assembly while also using the phosphonic acid moieties as a *H*-bonding site. Thus, we hypothesise that these hydroxyl groups of phosphonic acid of NDIs with melamine (MM) will be beneficial for controlled assembly *via* H-bonding, as MM acts very well as a proton donor in self-assembly process^13^,^18^. Here, we describe a novel flower-shaped morphology based on hierarchically organized supramolecular nano-to-microarchitecture that is readily prepared by self-organization of phosphonic acid appended NDI with MM in water.

## Results

**Figure 1:**
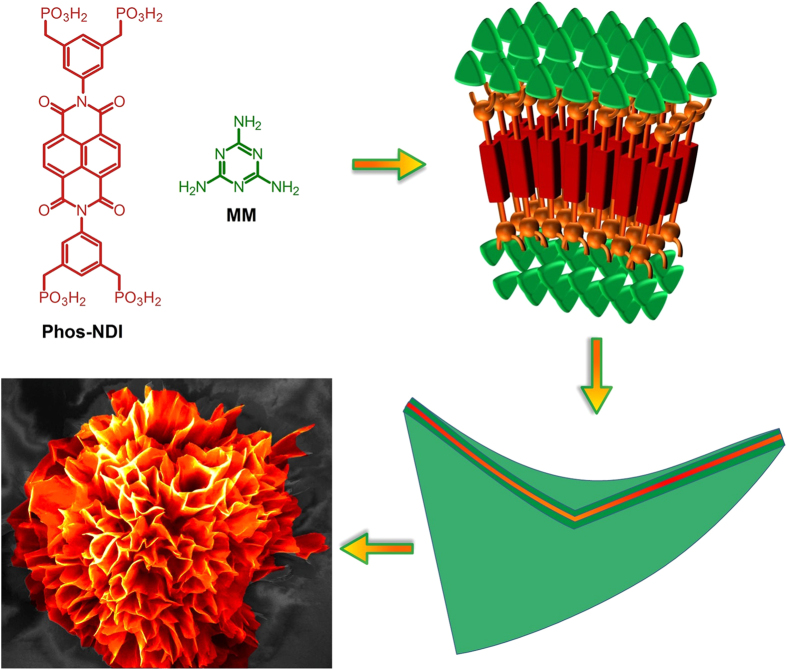
Schematic diagram illustrating flower-like assembly of Phos-NDI **1** with melamine (MM).

Flower-shaped morphologies of crystallized mineral salts (calcium sulphate) are seen in nature^46^ and often even in inorganic material systems^19^,^20^,^21^,^22^,^23^, very rare in organic^24^,^25^,^26^,^27^,^28^,^29^,^30^,^31^ material but never happen in water due to their solubility properties. To the best of our knowledge, the flower-shaped morphology forming in aqueous medium described herein is the first example of such assembly being observed in a supramolecular system containing two organic components (NDI appended phosphonic acid (Phos-NDI) and melamine (MM)). In this case, phosphonic acid-substituted at imide position of NDIs (**1**) possess two important properties resulting in the formation of controlled flower-like nanostructures: (i) the aromatic core of the NDI which is designed to optimize the dispersive interactions (π**-**π**-**stacking and van der Waals interactions) between the cores within a construct and (ii) phosphonic acid of NDI interact with MM through strong hydrogen-bonding (H-bonding). We believe such arrangements prevent crystallization and favour the directional growth of flower-like nanostructure in 3D fashion as illustrated in Fig. 1. The flower-shaped assembly formation was visualised by scanning electron microscope (SEM) and transmission electron microscopy (TEM) imaging, furthermore, XRD and DLS were used to determine the mode of aggregation.

A Phos-NDI bolaamphiphile **1** was synthesized in two steps from commercially available 1,4,5,8-naphthalenetetracarboxylic dianhydride (NDA) as outline in ESI Fig. 1. In the first step the suspension NDA and phosphonated aniline in dry *N, N’-*dimethyl acetamide (DMA) was heated at 120 °C, yielded **2** as brown semisolid in 60%. In the next step Phos-NDI bolaamphiphile **1** was achieved in 69% as white solid from **2** *via* deprotection of the phosphonate esters of **1** using TMSBr in dry acetonitrile at 0 °C to 40 °C followed by protonation using methanol, and unambiguously characterised^45^.

### UV-vis absorption and fluorescence spectroscopy

**Figure 2:**
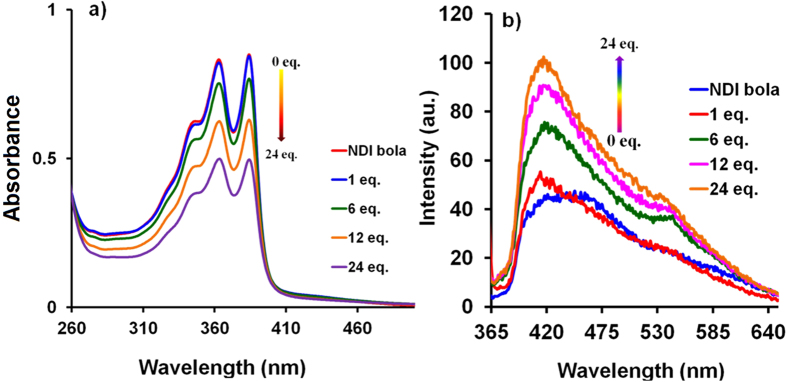
Solution based self-assembly. (**a**,**b**) UV-vis absorption and emission changes of Phos-NDI **1** (0.5 × 10^−5^ M) upon adding 0–24 equiv. of MM (1 × 10^−2^ M) in water, respectively.

Phos-NDI bolaamphiphile **1** is water soluble due to the presence of four phosphonic acid groups. Typically, supramolecular self-assembly flower-like assembly was prepared by mixing of the solution of Phos-NDI **1** in water with the solution of MM in water at varying ratios between both the components. The UV-vis absorption of **1** (0.5 × 10^−5^ M) in water showed typical vibronically saturated spectra with two well resolved sharp absorption peaks at 363 nm and 384 nm along with a shoulder at 347 nm, which is characteristic of the S0→S1 transition (Fig. 2a). It can be clearly seen that incremental addition of MM (0–24 equiv.) resulted in a reduction in peak intensity due to enhanced aggregated structure formation of **1** incorporation with MM. Furthermore, Fig. 2a shows the absorption spectra of **1** with an incremental addition of MM which clearly shows a reduction of absorption peak intensity. Fluorescence spectroscopy provides evidence for aggregation of **1**, depending on the concentration of MM used (Fig. 2b). Compound **1** shows emission at maxima at 439 nm (λ_ex_ = 384 nm) in water, which is blue-shifted to 415 nm (~24 nm) upon incremental addition of MM. This suggests that the mode of aggregation varies depending on the concentration of MM used. Nevertheless, when more than 24 equiv. of MM is used, the compound precipitates. The absorption and emission spectroscopy suggests formation of aggregates *via* H-bonding as well as face-to-face π-stacks of NDI chromophores in a similar effect observed for the case of J- and H-aggregates in similar assemblies^16^.

### Field Emission Scanning Electron Microscopy

**Figure 3:**
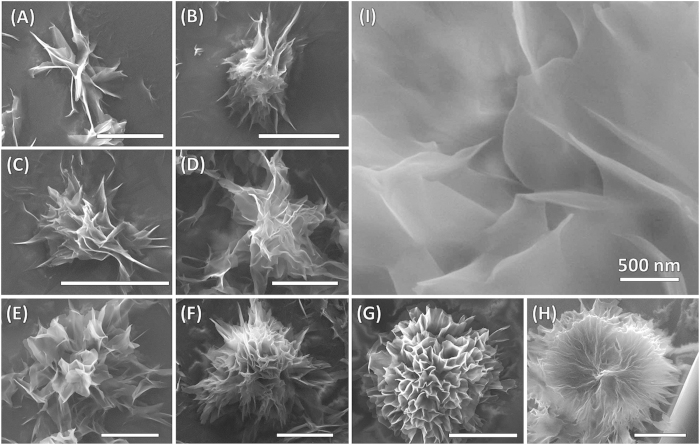
Visualisation of flowers by SEM analysis. (**A**–**H**) Field emission scanning electron micrographs (FE-SEM) of the 3D hierarchical flower like aggregate at various stages of growth during solvent evaporation. These aggregates formed from 1:12 molar ratio of Phos-NDI **1** and MM by mixing 1 × 10^−4^ M of Phos-NDI **1** and 5 × 10^−3^ M of MM solutions. These fractal structures consist of interweaved micosheets (the scale bars are 5 μm), and (**I**) A zoomed image showing fractal structures consist of interweaved micro-sheets.

To gain further insight, we performed Field Emission Scanning Electron Microscopy (FE-SEM). The microstructures produced upon water evaporation of Phos-NDI **1** and MM and mixes (1:1, 1:6, 1:12 and 1:24 *molar ratio*) of these two constituents facilely by drop-casting on silicon wafer substrate. Typically, larger 3D hierarchical flower-like aggregate formed from 1:12 and 1:24 molar ratio of Phos-NDI **1** and MM by mixing 1 × 10^−4^ M and 5 × 10^−3^ M solutions, respectively. It can be noted that a flowerlike morphology several micrometers in size (5–10 μm) with flake-like nanostructures several nanometers in thickness were observed (Fig. 3A–H). Solutions of MM and Phos-NDI **1** were mixed at various ratios and allowed to reach equilibrium for three hours and subsequently air dried on the substrate leaving flower-like superstructure on the surface. The formation of these super structures at the aforementioned ratios highlights the delicate balance of the multiple hydrogen bonding donor and acceptor sites along with π-stacking of aromatic plane Phos-NDI bolaamphiphile **1**, allowing molecules to form supramolecular assemblies with MM *via H*-binding and π**-**π interactions, allowing nanostructures to form in a controlled 3D fashion. A zoomed-in SEM image (Fig. 3I) clearly reveals that the flowerlike assembly consists of wrinkled plate interweaved micro-sheets with several tens of nanometres in thickness. These fractal structures consist of wrinkled plate nanostructures.

**Figure 4:**
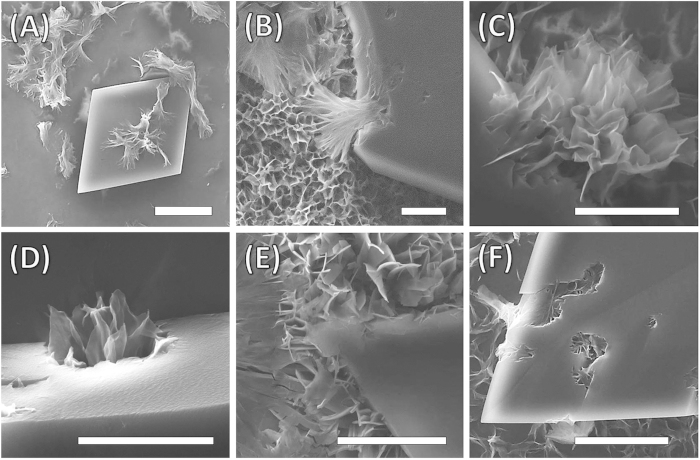
Flower assembly formation process. SEM images showing the formation process of the flower-shaped microstructure. (**A**–**D**) Intermediates of incomplete microflowers were observed within 3 h, which was in a quite similar manner mimicking the natural flowers blooming from blooms. (**E**,**F**) only the fully unfolded microflowers were observed after 3 h. The scale bar is 5 μM, respectively.

The addition of Phos-NDI **1** to MM solution at high ratios (1:24 and higher), where MM crystal formation by solvent evaporation is dominant, this shows the Phos-NDI **1** eroding the MM micro-crystallites resulting in the formation and immergence of flower like superstructure from crystals surface as seen in Fig. 4. The samples were prepared by water evaporation over 3 hours and the images taken were of formations at various stages of growth of the same sample. The flower like structure is a three dimensional hierarchically assembled supramolecular fractal architecture formed from non-planar two dimensional sheets, which can collapse to form crystalline solid when the substrate contain large number of overlapping flower structures (see Supplementary Information Figure S2). The sheets forming the flower like aggregates are more dispersed in less populous regions of the substrate followed solvent evaporation.

It is important to mention that the organised flower-like morphologies of **1** and MM are dependent upon the mixing ratios of these two components (See Fig. 3). In order to gain insight into the intermolecular interactions at the molecule-level, we performed FE-SEM study of **1** and MM at 1:1 and 1:6 molar ratios. The self-assembled aggregation arrangement 1:1 and 1:6 molar ratio of Phos-NDI **1** and MM produced randomly deposited irregular sheet with small fractal pattern like supramolecular structures (B) that yet to form more complex fractal structures as seen in the higher ratios of MM shown in (C), (D) and (E). However, the Phos-NDI **1** alone did not produce crystalline or fractal flower like structures but a large scale order of tubular formation forming a blueprint of a rhombic structure as shown (A) MM formed regular rhombic crystalline structures (F) and no smaller microstructures observed as shown in the Supporting Information Figure S3.

To evaluate the effect of the substrate hydrophilic/hydrophobic properties on the self-assembly of Phos-NDI/melamine is studied at 1:12 molar ratio using functionalised glass substrate. Glass substrates were washed using 1 N KOH to produce a hydrophilic surface and silanized using 2% TMS chloride in heptane for 3 hours to produce a hydrophobic surface. Furthermore, the contact angle is ~10° for the hydrophilic and ~91° for the hydrophobized surfaces, however, the contact angle for the silicon wafer surface after acetone, ethanol and then Milli Q water wash was 47° determined (Supplementary Information Figure S4). SEM micrographs from highly hydrophobic surface (Figure S4a–c) and hydrophilic surface are shown in Supplementary Information Figure S5d,e. It can be clearly seen that the fundamental features of the self-assembly i.e. bend flakes are forming on both hydrophilic and hydrophobic substrates. Typically, on hydrophobic surface water droplets spread very thin and leaves more dispersed self-assembled structures. While on hydrophilic surface solvent evaporation is very slow, thus, formation of dense flower-like superstructures is observed.

### Transmission electron microscopy

**Figure 5:**
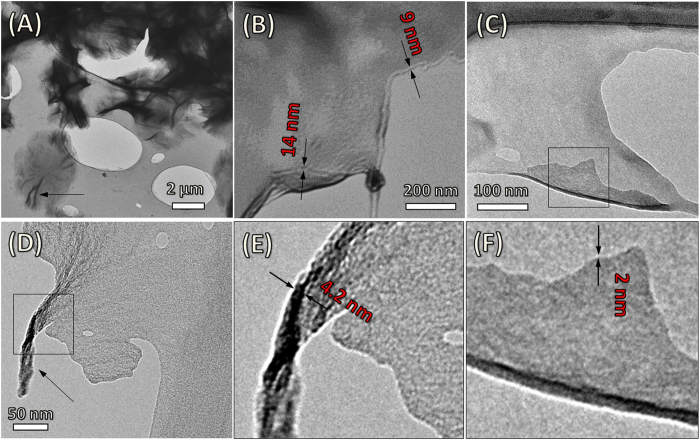
Transmission electron microscopy analysis. TEM images of thin film of Phos-NDI **1**– MM at 1:12 ratio. TEM images show molecularly thick sheets (down to 4.2 to 2 nm as in E and F, higher thicknesses are more abundantly observed as seen in (**B**) that twist and spin at the edges (**D**) to form the flower like structure. The sheets crease and bend in the middle to produce the 3D formation (**A**).

In order to evaluate the defined assembled structure of the flowerlike assembly on nanometre scale, transmission electron microscopy (TEM) investigation was performed. TEM images of thin film of Phos-NDI **1** and MM at 1:12 ratio were measured. TEM images show nano-thin sheets (down to 4.2–2 nm nm, Fig. 5E,F) with curvature twisting at the edges (D) to form the flower like structure, which is in good agreement with the single bilayer distance estimated from the two component assembly (NDI and MM). Further growth of these sheets increased the sheets thickness as in (B) proceed to form a 3D fractal superstructure (Fig. 5), which support formation of microflower-like assemblies as seen in the SEM micrographs (Fig. 3). The molecular thickness of these sheets is indicative of molecular arrangements that do not allow further crystal growth in the z-direction to occur. Considering that these superstructures are dominated by the MM component and the strong π**-**π**-**stacking between the NDI cores, the molecularly packed plane (or membrane) must curve to accommodate for the large number of MM molecules on the surface.

### XRD diffraction and DLS study

**Figure 6:**
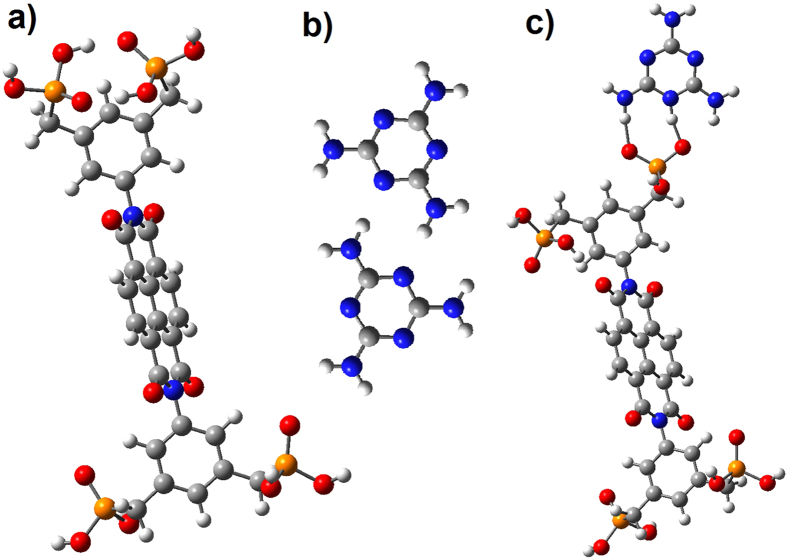
Theoretical density functional theory (DFT) calculations shows H-bonding between: a, NDI-NDI, b, M-M and c, NDI-MM, respectively.

**Figure 7:**
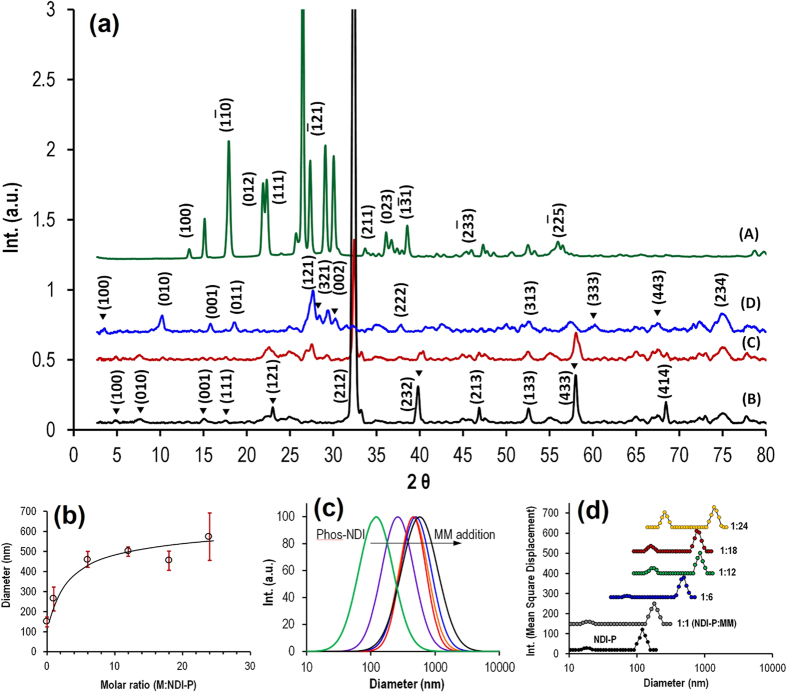
(**a**) Powder X-ray diffraction pattern of (A) melamine, (B) Phos-NDI **1**, (C) Phos-NDI **1**- MM at 3:8, and (D) 1:8 ratios. B, C, and D are scaled by 3 to show the significance of minor reflections. (**b**) Average hydrodynamic diameter of aqueous Phos-NDI aggregates and the increase of their size with MM addition up to 1:24 molar ratio measured using dynamic light scattering (DLS) particle size analyser. (**c**) Log Normal distribution. (**d**) Mean square displacement changes starting from Phos-NDI followed by the addition of melamine.

To understand growth the powder XRD diffraction pattern was used (Fig. 6a), obtained results of MM is in agreement with literature as described by Hughes group^47^. The XRD pattern was indexed to monoclinic unit cell with *P2*_*1*_*/a* symmetry and the calculated lattice constants are *a*_*o*_ = 10.54 Å, *b*_*o*_ = 7.45 Å, *c*_*o*_ = 7.25 Å, and β = 112° 2′. The MM crystallises as planar sheets of hydrogen bonded molecules. The XRD pattern of Phos-NDI **1** obtained by GADDS (general area diffraction detection system) can be indexed to orthorhombic system with unit cell parameters of *a*_*o*_ = 18.22 Å, *b*_*o*_ = 11.56 Å, and *c*_*o*_ = 5.91 Å. The lattice constants are within the size range of the molecule and indicative of periodic alignment of Phos-NDI **1** molecules along the <212> direction assigned to the 2θ = 32.4°. This is in agreement with π-π stacking of NDI cores due to the dominant π-π interaction, resulting in a preferential growth perpendicular to the position of the molecule plane within the unit cell. When Phos-NDI **1** is mixed with melamine, the size of crystallite domains was found to decrease significantly and the resulting phase becomes increasingly amorphous. Hydrogen bonding and π-π interaction determine the crystal structure of Phos-NDI **1**, therefore while the π-π interaction dominate the stacking of the molecules perpendicular to the NDI core plane H-bonding determine the in plane growth of sheets. These H-bonds are broken when in contact with MM to form a stronger one between the acidic phosphonate and the basic amine moieties. The ratio of MM to Phos-NDI **1** determines the energy balance between the broken bonds in the pure compounds and the newly formed bonds between Phos-NDI **1** and MM. The sheets are only formed when the interaction between Phos-NDI **1** molecules is mainly hydrophobic π-π and between Phos-NDI **1** and MM is mainly H-bonding. This reduces the Phos-NDI **1** to a single sheet of sandwiched between two layers of hydrogen bonded MM molecules. The SEM and TEM images show sheets 2–3 nm in thickness which is within the dimensions of a single Phos-NDI **1** molecule and a MM molecule bound to the phosphonate group *via* H-bonds (Fig. 7). The steric hindrance of the large phosphonate moieties causes a twist and folding in these sheets that is the main reason for the 3D fractal growth to form the flower like structure. The sheets of MM molecules sandwiching the Phos-NDI **1** layer can increase in thickness with higher MM ratios resulting in a more defined and self-supporting 3D supramolecular structures. At MM to Phos-NDI **1** molecular ratio of 8:3 the XRD diffraction is still dominated by the Phos-NDI **1** pattern indicative of incomplete dissolution of the MM phase in to the Phos-NDI **1** phase and the interactions happening only on the surface of the supramolecular Phos-NDI **1** sheets resulting in the significant reduction of the sheets dimensions, where sheets are separated by more and more MM molecules skirting their edges. This phenomena is evidenced by the intensity reduction of the <212> diffraction peak of Phos-NDI **1**. The *<hkl>* indexing of the powder pattern confirms the presence of Phos-NDI **1** as major phase together with undissolved MM existing as a second phase.

However, when the MM molecular ratio in the mixture is increased to 8:1, the XRD diffraction pattern showed that the peaks originating from Phos-NDI **1** crystal phase were absent and new reflections appear for this composition indicative of formation of new unit cell structure. The *<hkl>* indexing of the diffraction peaks for this molecular ratio showed the formation of a new triclinic phase with lattice constants of *a*_*o*_ = 27.80 Å, *b*_*o*_ = 8.74 Å, *c*_*o*_ = 6.36 Å, and α = 96°16′ β = 113° 36′, γ = 69° 36′. The main basal reflection has completely disappeared, this is evidence of π-π stacking disappearing in the supramolecular arrangement, the new structure is reliant on the charge interaction and hydrogen bonding which formed on the edges of the Phos-NDI **1** and MM molecules not by the molecular plane. The strong interaction between the phosphonate groups and MM are similar to those of phosphate ion and MM discussed by Huang *et al.*^48^, therefore the molecular arraignment between Phos-NDI **1** and MM cannot maintain the co-planarity of MM molecules and Phos-NDI **1** molecules. The new arrangement results in twisted sheets on the large scale but maintain a low crystallinity with a specific unit cell on the atomic scale of the material. The SEM and TEM images of high ratio of MM to Phos-NDI **1** show a film of monolayer of such arrangement.

Furthermore, DLS analysis shows hydrodynamic diameter larger than the pore size of the cellulose acetate filter used to remove large particles from the MM solution (Fig. 6b–d). This can be an indicative of the dynamic interaction in of the molecules in the supper structure in the solution. The addition of the Phos-NDI **1** has increased this average hydrodynamic diameter to ~1.5 μm at 2:1 ratio of Phos-NDI **1** to melamine. Initially the diameter decreases at higher ratios of MM to Phos-NDI **1** breaking of the initial aggregates and the formation of new mixed aggregates, these aggregates then grow with the addition of more Phos-NDI **1**. Dynamic laser scattering study of the aggregation of MM and Phos-NDI in aqueous solution was conducted by measuring the mixture of the components filtered saturated solutions. The initial Phos-NDI showed aggregates with an average hydrodynamic diameter of 148 ± 25 nm. The addition of the MM solution to form a molar ratio 1:1 to 1:24 has increased the average hydrodynamic diameter to 460 ± 40 nm at 1:6 Phos-NDI to MM molar ratio, aggregate size has reaches equilibrium of 574 ± 118 nm at 1:24 molar ratio. The size of these aggregates is much smaller than the flower like superstructures observed using SEM and TEM microscopy. The flower like superstructures is formed by the assembly of these aggregates during solvent evaporation.

We hypothesise that; such flower-like assemblies are mainly derived by *H*-bonding of the phosphonic acid of NDI and melamine along with π**-**π interactions within the core of the NDI molecules. As the p*K*_a_ values of -H_2_PO_3_ are p*K*_a1_ = 2.16, p*K*_a2_ = 7.21, and p*K*_a3_ = 12.32. It is well known that 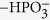 species reach a plateau and drop when pH decreases <6.0, thus, 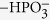 species is greater than 94% in the range of pH 4–6. This indicates that 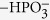 is a more important reactant than other phosphonic acid species 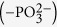
49. On the other hand, MM is weak base with three different p*K*_a_ i.e. p*K*_a1_ = 5.10, p*K*_a2_ = 0.20, and p*K*_a3_ = −2.10, respectively^50^. MM has two main species in the pH 4–6: MM and HMM^+^. Thus, with increasing pH the conc. of HMM^+^ decreases. Therefore, the most possible reactants in flower assembly is 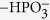, MM and HMM^+^ in the pH range, which have a remarkable effect on the electrostatic and hydrogen-bonding interactions among the reactants.


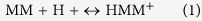



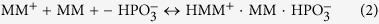


### Theoretical density functional theory

Theoretical density functional theory (DFT) calculations with no consideration of dispersion interactions in gas phase using Gaussian 09 suite of programs and B3LYP/6-31G level of theory of Phos-NDI **1** and melamine and the interaction between these two molecules shows that the H-bonds between these two molecules are ~9 kcal/mol more stable than the H-bonds within these molecules (Fig. 7)^51^. The results of these calculations shows −6.2 and −6.7 kcal/mol energy for H-bonds within a Phos-NDIs and in a dimer of melamine respectively, which are within the range of strength of typical H-bond. Multiple H-bonds in a self-assemble molecular structure can increase H-bonds stability. Here the H-bonds between Phos-NDI and melamine (Fig. 7c) show a significant increase in stability reaching −15.8 kcal/mol. The obtained values here are for interaction between individual gas phase molecules, larger self-assemblies result in further enhanced energy stability. This reveals the driving force behind the formation of these flower-like supramolecular structures.

## Discussion

We described formation of micrometer-sized flower-shaped supramolecular structures, which were spontaneously and quantitatively obtained by hierarchical self-assembly of a two components (NDI bearing phosphonic acid and melamine) in aqueous medium for the first time. Our approach to the hierarchical organization of the derivative utilizes π**-**π and *H*-bonding interactions of phospnonic acid NDI and melamine. In which hydroxyl-functional group of phosphonic acid NDI act as a proton donor and melamine act as a proton acceptor. Variation of the ratios of these two components gives step-wise growth mechanism of assembly, as it can be seen that NDI to MM ratio 1:1 and 1:6 produces shorter flakes intermediates, however, at higher ratios such as 1:12 and 1:24 flower-like structures are produced. The formed flower-structures were characterised by SEM, TEM, DLS and XRD analysis. In general, the experimental results from DLS, SEM, suggested a two-staged mechanism, as the growth of flower was started with wrinkled plate interweaved micro-sheets with several tens of nanometres in thickness which then transformed into microscopic flower-shaped objects. Therefore, to form such morphology, first the non-specific H-bonds hold fast the individual molecules together to form the nucleation and grow into wrinkled plates. Once they are held closer a reorganizing process follows to produce flower-like structures. Importantly, the ability to self-organize well-defined and discrete flower-like objects with complex morphologies may stimulate further advances in component design and self-assembly theory that go beyond simple morphologies.

In summary, the two components self-assembly flower-shaped supramolecular structure was revealed by various techniques. The self-assembly is mainly driven by hydrogen bond networks have been revealed by XRD analysis and DFT calculation. Therefore, phosphonate functional groups can also play an important role, with the combination of the rich chemistry of both proton donor and proton acceptor and its 3D conformational, to construct more complex chemical designs in the supramolecular self-assembly area. The results presented herein demonstrate an actionable roadmap to pursue may stimulate further advances in component design and self-assembly theory that go beyond simple morphologies.

## Materials and Methods

### Material and measurements

Naphthalenetetracarboxy dianhydride, melamine (MM), acetic acid (AcOH), chloroform (CHCl_3_), chloroform-d (CDCl_3_), methanol (MeOH), dichloromethane (DCM), were purchased from Aldrich and used without purification, unless otherwise specified. Fluorescence measurements were performed on a FluoroMax-4, Horiba Jobin Yvon, equipped with an injector port, a stirrer and a temperature controller (25 °C). ^1^H NMR, ^13^C-NMR spectra were recorded on a Bruker spectrometer using CDCl_3_ as solvent and tetramethylsilane as an internal standard. The solvents for spectroscopic studies were of spectroscopic grade and used without further purification.

#### Sample preparation

Stock solutions of phosphonate substituted naphthalene diimides **1** (c = 1 × 10^−4^) were prepared in Milli-Q water. A 0.2 mL aliquot of the stock solution of each one was transferred separately to four different volumetric flasks containing melamine (c = 5 × 10^−3^) with the ratio of 1:1, 1:6, 1:12 and 1:24, respectively, and made up to 2 mL volume with respective solvents. The solutions were allowed to equilibrate for 2 h prior to the Scanning Electron Microscopy (SEM) measurements.

### Spectroscopic measurements

#### UV-Visible Measurements

UV-vis absorption spectra were recorded in a Cary-50. UV-vis spectrometer in 1cm path length cuvette. The solutions were allowed to equilibrate at room temp for 2 h before spectral measurements.

#### Fluorescence Measurements

Fluorescence emission spectra were recorded in a Horiba Jobin Yvon FluoroMax®-4–Spectrofluorometer. Fluorescence measurements and quenching experiments were performed on a FluoroMax-4 equipped with an injector port and stirrer at 25 °C. All experiments were performed in a quartz cell with a 1 cm path length with 384 nm excitation wavelength.

### SEM imagining

The silicon wafer was cleaned by acetone, ethanol and then Milli Q water. SEM Samples were prepared by solvent evaporation on a silicon wafer and then sputter coated with gold for 10 s at 0.016 mA Ar plasma (SPI, West Chester, USA) for SEM imaging using a FEI Nova NanoSEM (Hillsboro, USA) operating at high vacuum.

### TEM imagining

TEM samples were prepared by solvent evaporation on a holey carbon grid and micrographs were produced using a Jole 1010 100 kV Transmission Electron Microscope.

### Molecular modelling

Density functional theory (DFT) calculations with no consideration of dispersion interactions in gas phase were conducted using Gaussian 09 suite of programs. Hydrogen bond energies were estimated by comparing the total energy of structurally minimized molecules and assembled molecules with H-bonds (a dimer of melamine molecules with N–H ….. N, Phos-NDI with internal H-bonds, and a melamine–Phos-NDI assembly with P = O ….. N–H and P–O–H ….. N).

## Additional Information

**How to cite this article**: Bhosale, R. S. *et al.* Flower-like supramolecular self-assembly of phosphonic acid appended naphthalene diimide and melamine. *Sci. Rep.* **5**, 14609; doi: 10.1038/srep14609 (2015).

## Supplementary Material

Supplementary Information
