# Supplementary material for: Flower-like supramolecular self-assembly of phosphonic acid appended naphthalene diimide and melamine
Source: Sci Rep. 2015 Sep 29;5:14609. doi: 10.1038/srep14609 (PMC4586721; doi:10.1038/srep14609)
Supplement: Supplementary Information [file srep14609-s1.pdf]

Supplementary information

Flower-like supramolecular self-assembly of phosphonic acid appended naphthalene diimide and melamine

Rajesh S Bhosale,[1] Mohammad Al Kobaisi,[2] Sidhanath V. Bhosale,[3] Suresh Bhargava,[2,4] Sheshanath V. Bhosale[2,*]

[1]RMIT-IICT Research Centre, CSIR-Indian Institute of Chemical Technology, Hyderabad- 500 007, Telangana, India. [2]School of Applied Sciences, RMIT University, GPO Box 2476, Melbourne, Vic. 3001, Australia. [3]Polymers and Functional Materials Division, CSIR-Indian Institute of Chemical Technology, Hyderabad-500 007, Telangana, India. [4]Centre for Advanced Materials and Industrial Chemistry (CAMIC), RMIT University, GPO Box 2476, Melbourne, Vic. 3001, Australia.

Corresponding authors: Tel.:+61399252680; E-mail: sheshanath.bhosale@rmit.edu.au

Synthesis of Phos-NDI bolaamphiphile **1.** Compound **1** was synthesised following literature protocol.[S1]

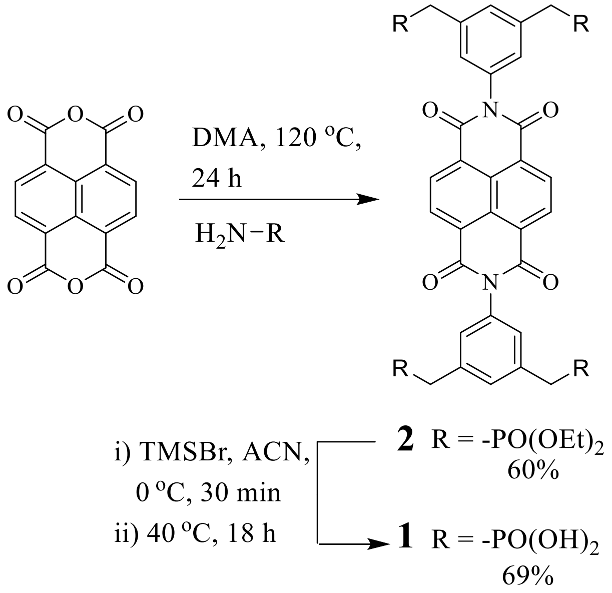

Figure S1 | Synthesis of Phos-NDI bolaamphiphile **1**.

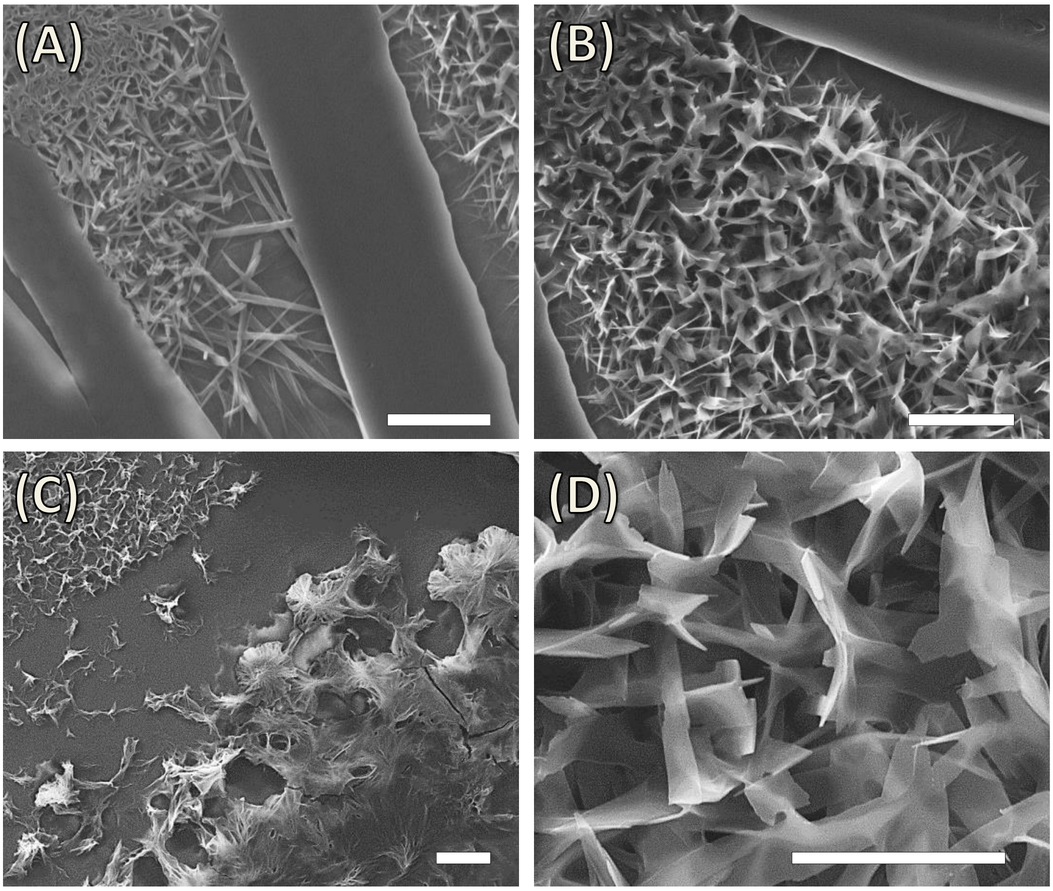

Figure S2 | At high ratios of 1:24 and higher of Phos-NDI to MM, the sheets forming the flower like aggregates are more dispersed in less populous region of the substrate followed solvent evaporation. Scale bar is 10 μm in (A, B, C) and 5 μm in (D). These sheets have collapsed to form crystals as shown in (A) without passing through a flower like fractal structure.

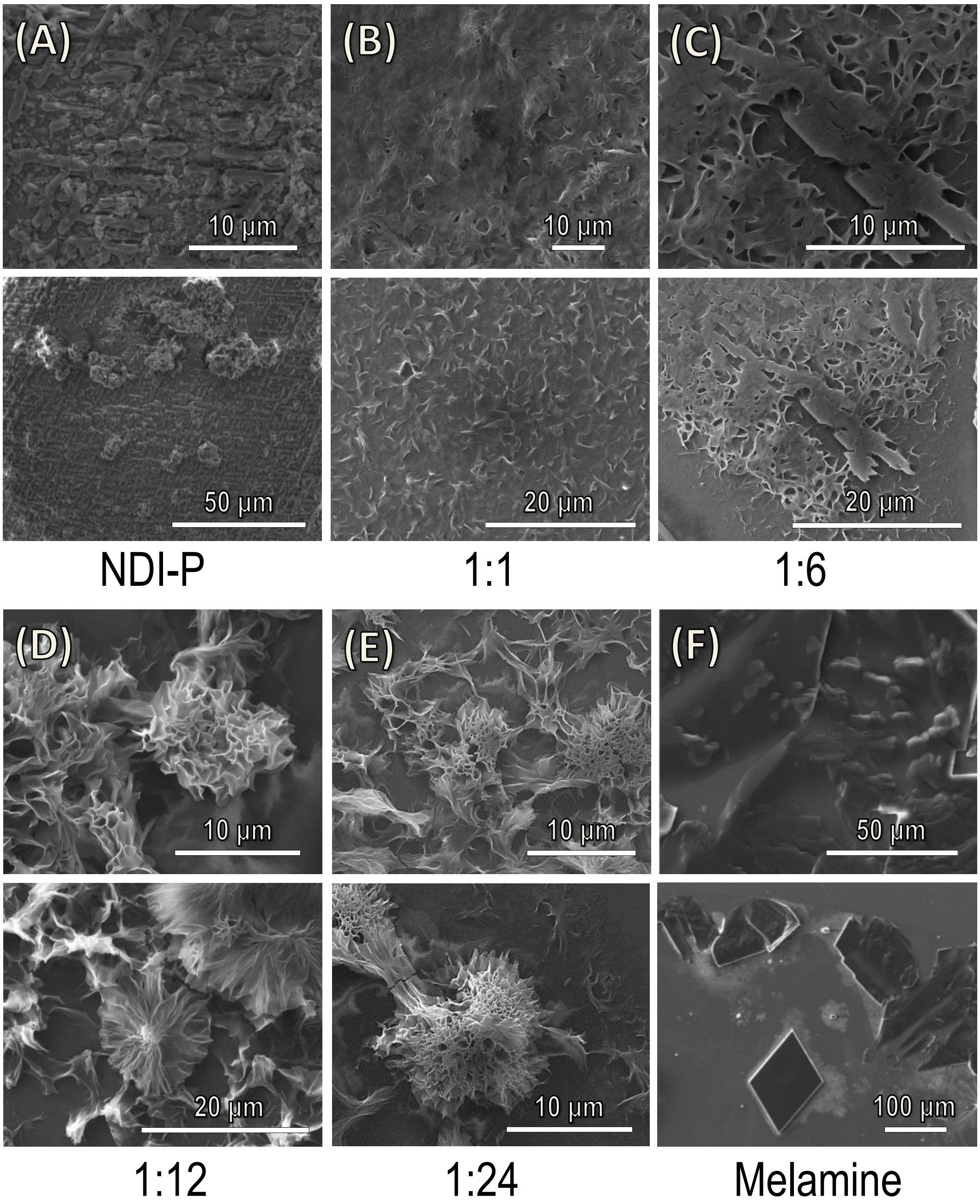

Figure S3 | The microstructures produced upon water evaporation of Phos-NDI and MMand mixes of these two constituents. The Phos-NDI solution did not produce crystalline or fractal flower like structures but a large scale order of tubular formation forming a blueprint of a rhombic structure as shown (A). MM formed regular rhombic crystalline structures (F) and no smaller microstructures observed. The 1:1 molar ratio of Phos-NDI to MM produced randomly deposited irregular sheet like supramolecular structures (B) that yet to form more complex fractal structures as seen in the higher ratios of MM shown in (C), (D) and (E).

Glass substrates were washed using 1 N KOH to produce a hydrophilic surface and silanized using 2% TMS chloride in heptane for 3 hours to produce a hydrophobic surface. The contact angle is ~10° for the hydrophilic and ~91° for the hydrophobized surfaces. The contact angle for the silicon wafer surface after acetone, ethanol and then Milli Q water wash was 47°.

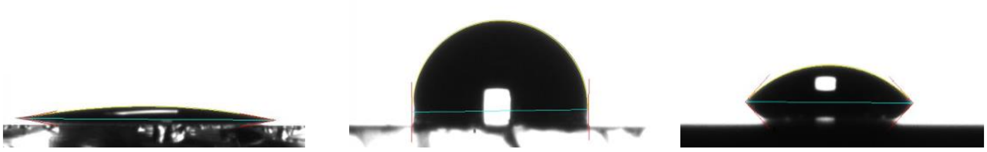

Contact angle	11.62°	91.32°	47.70°
Base width	4.14 mm	3.44 mm	2.71 mm
	KOH treated glass	TMS glass	cleaned Si wafer

Figure S4 | The contact angle for the hydrophilic glass, hydrophobic glass and silicon wafer surface.

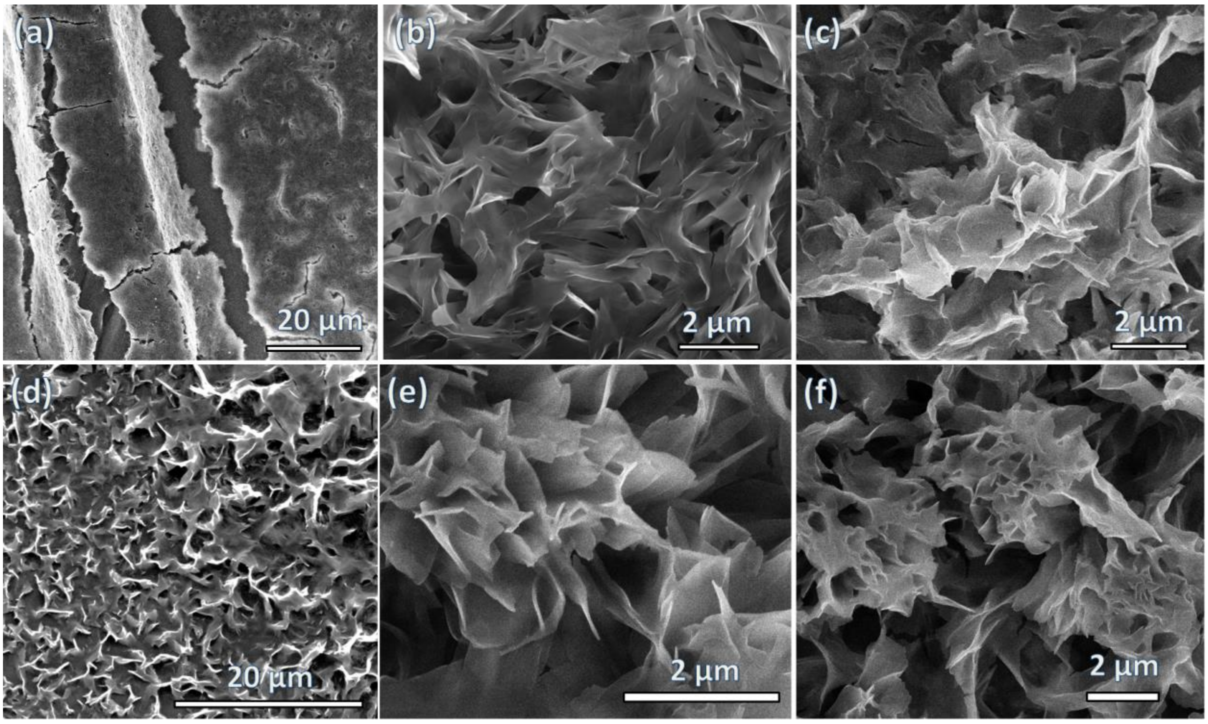

Figure S5 | The SEM microstructures produced upon water evaporation of Phos-NDI and MM (1:12, v/v): (a-c) on hydrophobic glass substrate and (d-f) on hydrophilic glass substrate.

XRD Spectroscopy: These measurements were carried out on a Bruker D8 Discover micro diffraction system with general area detector diffraction system (GADDS) instrument operating at a voltage of 40 kV and a current of 40 mA with Cu Kα radiation. Samples were deposited from water solutions on glass coverslips, and 40 minutes time was used to accumulate the spectra of MM and 14 hours to accumulate the spectra of Phos-NDI **1**, and Phos-NDI **1** to MM ratios of 3:8 and 1:8. The <*hkl*> indexing of XRD patterns of based on GADDS measurements of Phos-NDI **1** and Phos-NDI/MM at 1:8 molecular ratios.

**Phos-NDI** 1			
parameter	value	sigma	95% conf
a	18.22	0.00	0.01
b	11.56	0.00	0.01
c	5.91	0.00	0.00
cell vol	1243.51	0.21	0.46

**Phos-NDI:M**	**(1:8)**		
**parameter**	value	sigma	95% conf
**a**	27.800	0.050	0.114
**b**	8.737	0.004	0.009
**c**	6.358	0.004	0.009
α	96.282	0.053	0.123
β	113.605	0.074	0.171
γ	69.605	0.078	0.180
**cell vol**	1325.492	2.371	5.472

DLS analysis: The particle size distribution of Phos-NDI **1**-MM aggregates in aqueous medium were studied using dynamic light scattering particle size analyzer (DLS) at various ratios were 5×10^{-4} M of Phos-NDI **1** and 5×10^{-3} M of MM were filtered using cellulose acetate 0.21 µm syringe filters and mixed at various volumetric ratios. DLS measurements were conducted using Brookhaven Instrument Corp., 90Plus Particle Size equipped with a He–Ne laser (632.8 nm, 35 mW) and quartz cuvette.

Table S1. DFT calculation of Phos-NDI hydrogen-bonding

	Phos-NDI : With no internal hydrogen bonding	Phos-NDI : With internal hydrogen bonding	Phos-NDI - MM	MM	2 MM
Calculation Type	FOPT	FOPT	FOPT	FOPT	FOPT
Calculation Method	RB3LYP	RB3LYP	RB3LYP	RB3LYP	RB3LYP
Basis Set	6-31G	6-31G	6-31G	6-31G	6-31G
Charge	0	0	0	0	0
Spin	Singlet	Singlet	Singlet	Singlet	Singlet
Total Energy (a.u.)	-3836.56570629	-3836.58544269	-4282.96038287	-446.34446380	-892.71042062
RMS Gradient Norm (a.u.)	0.00000291	0.00000291	0.00000268	0.00004419	0.00001088
Dipole Moment (Debye)	4.1884	4.7403	7.7676	0.0005	0.0006

	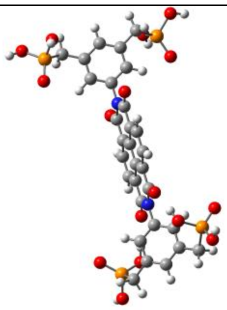	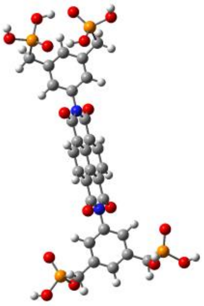	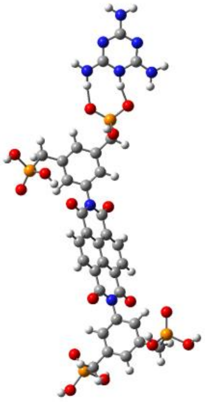	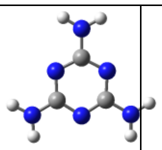	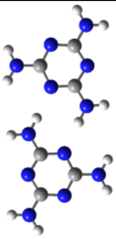

References

S1. Nandre, K. P., Bhosale, S. V., RamaKrishna, K. V. S., Gupta, A. & Bhosale, S. V. A phosphonic acid appended naphthalene diimide motif for self-assembly into tunable nanostructures through molecular recognition with arginine in water. *Chem. Commun.* ***49***, 5444-5446 (2013).
